# Nanowires: Exponential speedup in quantum computing

**DOI:** 10.1016/j.heliyon.2024.e31940

**Published:** 2024-05-25

**Authors:** Mariam Akter Mimona, Md Hosne Mobarak, Emtiuz Ahmed, Farzana Kamal, Mehedi Hasan

**Affiliations:** a Department of Computer Science & Engineering, IUBAT-International University of Business Agriculture and Technology, Bangladesh; b Department of Mechanical Engineering, IUBAT-International University of Business Agriculture and Technology, Bangladesh

**Keywords:** Nanowires, NWs, Quantum computing, Computation, Prospects, Challenges, Applications

## Abstract

This review paper examines the crucial role of nanowires in the field of quantum computing, highlighting their importance as versatile platforms for qubits and vital building blocks for creating fault-tolerant and scalable quantum information processing systems. Researchers are studying many categories of nanowires, including semiconductor, superconducting, and topological nanowires, to explore their distinct quantum features that play a role in creating various qubit designs. The paper explores the interdisciplinary character of quantum computing, combining the fields of quantum physics and materials science. This text highlights the significance of quantum gate operations in manipulating qubits for computation, thus creating the toolbox of quantum algorithms. The paper emphasizes the key research areas in quantum technology, such as entanglement engineering, quantum error correction, and a wide range of applications spanning from encryption to climate change modeling. The research highlights the importance of tackling difficulties related to decoding mitigation, error correction, and hardware scalability to fully utilize the transformative potential of quantum computing in scientific, technical, and computational fields.

## Abbreviation

NWs –: Nanowires
SNSPDs –: Superconducting-Nanowire Single-Photon Detectors
TIs –: Topological Insulators
TI –: Topological Insulator
TCI –: Topological Crystalline Insulator
SEM –: Scanning Electron Microscope
TEM –: Transmission Electron Microscopy
QD –: Quantum Dot
EDSR –: Electric-Dipole Spin Resonance
QED –: Quantum Electrodynamics
SC –: Superconducting
QEA –: Quantum-inspired Evolutionary Algorithm
QRG –: Quantum Rotation Gate
NISQ –: Noisy Intermediate-Scale Quantum
SNSPDs –: Superconducting Nanowire Single-Photon Detector
MF –: Majorana fermion
MZMs –: Majorana fermion zero modes
SPDC –: Spontaneous Parametric Down-Conversion
MZMs –: Majorana zero modes
QEC –: Quantum Error Correction
HHL –: Harrow, Hassidim, Lloyd
FTQC –: Fault-Tolerant Quantum Computation
QC –: Quantum Computing
QKD –: Quantum Key Distribution
NISQ: Noisy Intermediate Scale Quantum
QML –: Quantum Machine Learning
DL –: Discrete Logarithm
DLP –: Discrete Logarithm Problem
WSQSE –: Working Seminar on Quantum Software Engineering
SWEBOK –: Software Engineering Body of Knowledge

## 1 Introduction

Nanowires are a key component of quantum computing and are an example of the marriage of nanotechnology with quantum mechanics. These extremely thin structures, which are frequently only a few atoms broad, have special quantum characteristics that can be used to process information [1,2]. Qubits, the building blocks of quantum information, are transported through nanowires in quantum computing [3,4]. Their nanoscale size makes it possible to precisely regulate quantum states, which makes it easier to construct quantum gates and improves coherence [5,6]. To optimize quantum computer systems, researchers are investigating a variety of materials for nanowire production, including semiconductors and superconductors. The development of quantum systems' scalability and functionality is greatly aided by nanowires, which hold the potential to bring about revolutionary advances in computational power [7].

In quantum computing, nanowires are flexible platforms for qubits, and different methods are used for various materials. Utilizing electron charge states, semiconductor nanowires provide tunable characteristics for quantum information processing [8]. Using zero-resistance states, superconducting nanowires improve coherence and enable quantum entanglement [9]. Exotic particle statistics are used by topological nanowires to build reliable qubits with built-in fault tolerance [10]. Electron spin serves as the foundation for quantum information processing and storing in spin qubits within nanowires [11]. These many nanowire-based qubit architectures highlight how interdisciplinary quantum computing is, bringing together quantum physics and materials science to create fault-tolerant and scalable quantum information processing systems.

To manipulate qubits for computation, quantum gate operations are essential parts of quantum circuits. The Pauli gate modifies quantum states by applying particular rotations along the X, Y, or Z axes [12]. Phase gates contribute to quantum algorithms by introducing phase shifts [13]. Arbitrary rotations in the quantum state space are possible with rotation gates [14]. SWAP gates are necessary for the exchange of qubit states in quantum information transfer [15]. Together, these gates make up the quantum algorithm toolbox, which makes it easier to perform the complex manipulations needed for quantum computing.

A key area of research in quantum technology is entanglement engineering, which involves exact control over quantum states for use in quantum sensing and computing [[16], [17], [18]]. By adjusting electron spin states, quantum dots—nanoscale semiconductor structures—act as causes of entanglement [19]. Superconducting nanowires enhance quantum coherence by generating entanglement through the use of zero-resistance states [20]. Using exotic particle properties, topological nanowires provide robust entangled states that are essential for fault-tolerant quantum processing [21,22]. Long-distance entanglement transfer is made possible in quantum communication networks by photonic nanowires [23]. Entanglement helps quantum sensing by improving measurement accuracy [24]. This multidisciplinary discipline, which combines quantum physics and materials science, has the potential to develop quantum technologies with hitherto unheard-of capabilities.

The feasibility of quantum processing depends critically on quantum error correction, especially when topological qubits depend on unusual particles such as Majorana fermions [25,26]. These particles provide intrinsic fault tolerance by topological shielding against local faults via braiding operations [25]. To achieve quantum scalability, qubits must be coordinated in a hybrid system that combines different technologies, including topological qubits and superconducting circuits [27]. The issues caused by decoherence are addressed by braiding Majorana fermions, laying the groundwork for error-protected logical qubits [13,26,28]. The development of quantum scalability in conjunction with error correction solutions is imperative to fully realize the potential of quantum computing.

Applications and techniques for quantum computing span a wide range of industries. Quantum key distribution reduces the dangers associated with quantum assaults by ensuring safe communication channels in cryptography [29]. Quantum algorithms are useful for data analysis and machine learning, with the potential for increased computational efficiency with quantum machine learning methods [4,30]. Shor's technique presents a danger to traditional cryptography since it factorizes huge numbers quickly and affects discrete logarithm problems, which are important for maintaining cryptographic security [31]. On the other hand, Grover's approach influences optimization in data analysis by providing a quadratic speedup for unstructured search tasks [32]. These quantum algorithms and applications demonstrate how quantum computing has the potential to revolutionize a wide range of industries, including cybersecurity and computational optimization.

The emergence of quantum computing has significant ramifications for many industries. As quantum algorithms defeat traditional security methods, cryptography is going through fundamental transformations. Quantum simulations make it possible to precisely model intricate environmental processes to combat climate change [33]. Quantum computing also speeds up research on materials and optimization for renewable energy, which has the potential to completely change the energy industry [33]. The far-reaching effects include encouraging creative methods in environmental research and sustainable technology, as well as rethinking digital security [34].

Decoding mitigation, error correction, and hardware scalability are problems for quantum computing [25]. The development of fault-tolerant quantum systems, the investigation of new qubit technologies, and the optimization of quantum algorithms are the future directions [35,36]. To fully utilize quantum computing in a variety of scientific, technological, and computational domains, overcoming these obstacles is crucial.

## 2 Nanowire as qubits

Future electrical and optoelectronic devices are anticipated to heavily rely on nanowires (NWs), which present a novel approach for investigating phenomena at the nanoscale [37]. Nanowires are a one-dimensional example of a low-dimensional semiconductor material structure, which has become one of the most extensively researched areas of science and technology [38]. Nanowire technology advancements have transformed electronics by making it possible to build ultra-compact, high-performance transistors, opening the door for quicker, more energy-efficient electronic gadgets.

**Fig. 1:**
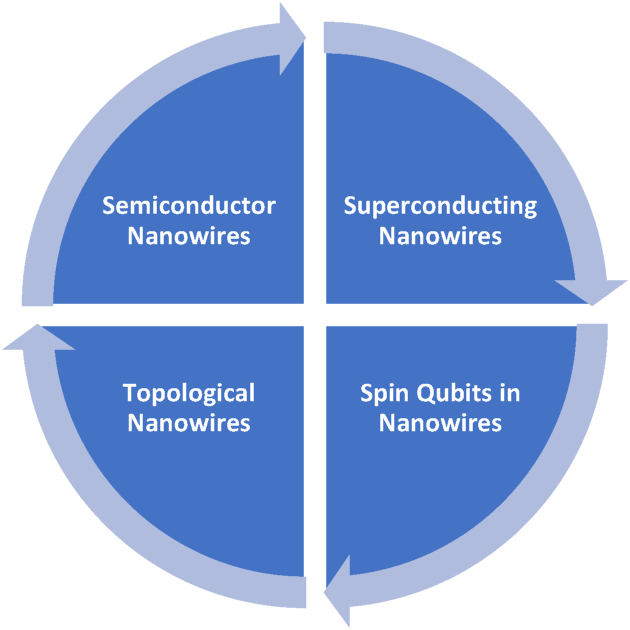
Nanowire as qubits.

There are two main strategies for generating 1-D nanoscale structures or NWs. First, there is the top-down method, which depends on using high-quality bulk beginning material from which it is feasible to remove certain materials and shape the final structure. There must be an additional advantage of converting the bulk material into a 1-D structure, resulting in increased functioning of the created material, for this procedure to be effective [39]. Additionally, it's crucial to manage the effects of etch damage on exposed surfaces since otherwise, this could reduce the inherent quality of the material. There are numerous instances in the literature where the top-down technique has been used to develop NW structures that improve device performance 8,9 and even demonstrate novel capabilities [40,41]. The only materials and material combinations that can be employed using this technology are those that are already available in bulk form [42]. Nanowires can be utilized in a variety of ways as qubits which are shown in Fig. 1.

### 2.1 Semiconductor nanowires

Semiconductor nanowires have demonstrated fascinating characteristics in the fields of nanophotonics, sensors, energy technologies, and advanced electronic devices that go beyond the current roadmap. There are multiple ways for fabricating nanowires, which can be categorized into three main groups [43].

1. Nanowires are formed by a process called bottom-up crystal formation, which is catalyzed locally.
2. Bulk crystals and epitaxial films are patterned and etched using a technique called top-down lithography.
3. Hybrid approaches combine elements from both categories 1 and 2.

The application of a gold metal catalyst enabled the vapor-liquid-solid growth mechanism, leading to the creation of silicon semiconductor nanowires with a one-dimensional, crystalline structure [44]. Hybrid systems that combine the macroscopic quantum features of superconductors with the capability to influence individual electrons have become a promising platform for investigating superconductivity [45]. Semiconductor nanowires have become a prominent area of research due to their significant contributions to the study of Majorana modes and topological superconductivity [46]. Majorana modes are quasiparticles characterized by their zero energy, which emerge in the vicinity of the border of a topological superconductor [47,48]. A number of investigations have provided evidence of electron transport in a semiconductor nanowire connected to a superconductor [[49], [50], [51], [52], [53], [54], [55]].

### 2.2 Superconducting nanowires

In recent times, there has been a rapid global expansion of interest in quantum technologies. The importance of systems for quantum communication, quantum encryption, and quantum key distribution has been specifically emphasized [[56], [57]]. Single-photons, being quantum entities, offer fascinating options for utilization as the medium in this technology [,^58^]. The primary options for adopting quantum technologies are single-photon detectors and nanoscale superconducting devices. The combination of solid-state and optical components forms superconducting-nanowire single-photon detectors (SNSPDs), which allow for high-speed (1.3 GBit s-1) quantum key distribution over extensive distances (>400 km), long-range (>1200 km), and space communication (239,000 miles) [59].

### 2.3 Topological nanowires

**Fig. 2:**
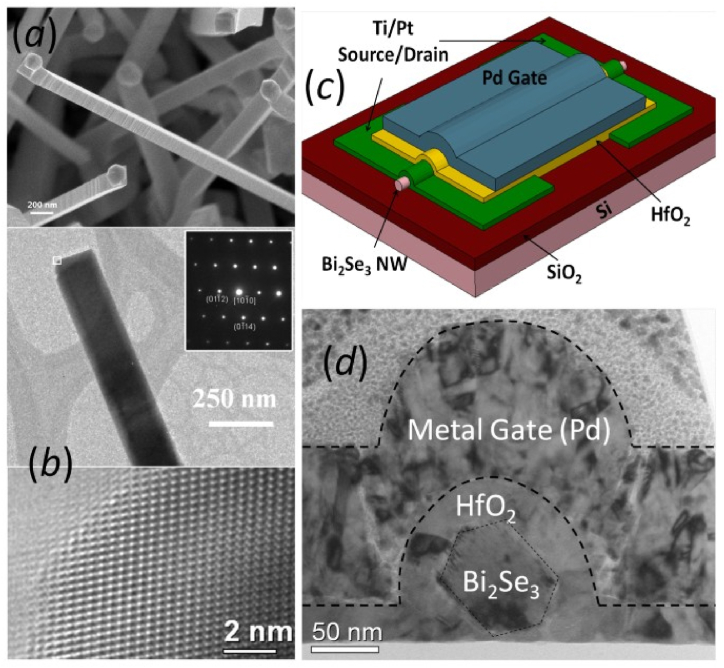
(a) A scanning electron microscope (SEM) image of the 10 m long, 50 nm–150 nm wide, as-produced nanowires. (b) The growth direction and single-crystal rhombohedral phase of the Bi2Se3 nanowires are identified. (c) depicts a schematic of the Bi2Se3 nanowire FET, and (d) depicts a TEM picture of the cross-section [70].

**Fig. 3:**
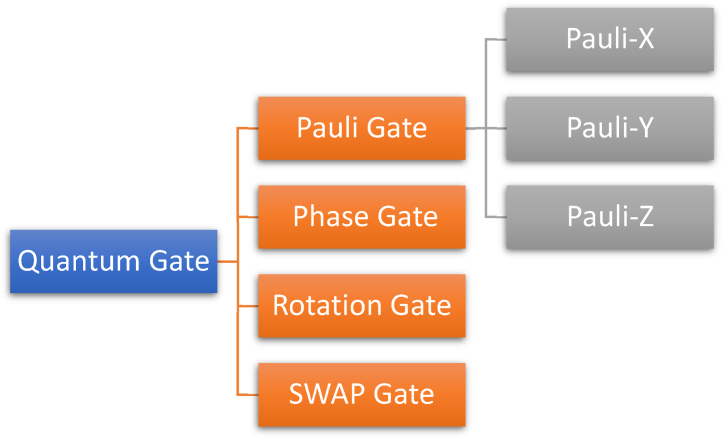
Quantum gates and their operations.

**Fig. 4:**
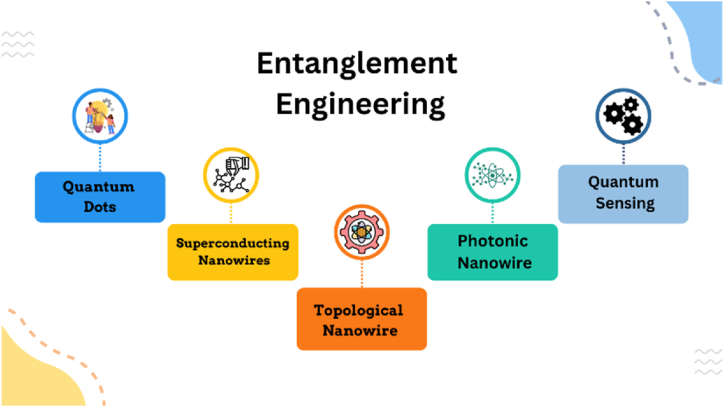
Quantum entanglement engineering techniques.

Over the past decade, there has been a rise in the study of topological materials, which are In recent years, there has been an increase in the investigation of topological materials, which are characterized by distinctive electronic band structures that deviate from those observed in conventional insulators and metals. This subject has emerged as a significant field of study in the field of condensed matter physics. At the interface of the materials, a complex band structure forms stable, spin-polarized electronic states that exhibit a linear correlation between energy and momentum. Accurate manipulation and dependable identification of topological states in nanostructures are crucial for maximizing their utility in electrical devices, given that the large surface-to-volume ratios of these structures can magnify the topological states. The number 64 is enclosed in square brackets. The earliest experimental discovery of topological materials, which can achieve a topological state without requiring intricate experimental conditions, occurred ten years ago [60]. Understanding the electrical band structure of topological materials, which have a unique band topology, is currently crucial for comprehending the physical properties of various materials [61]. Surface states in topological insulators (TIs) result from the inversion of the bulk bandgap, which is induced by the strong spin-orbit coupling of the heavy atoms present in the material. Time-reversal symmetry ensures the protection of these surface states. These materials have surface states that are safeguarded by topology and have distinctive electrical properties [62]. The work centered on analyzing the electron transport properties of nanodevices to understand the characteristics of the topological surface states in nanostructures [63]. Empirical data from multiple investigations on nanostructures of topological insulators (TI) and topological crystalline insulators (TCI) has conclusively demonstrated the existence of the expected topological surface states. These states demonstrate a helical Dirac characteristic. The number 69 is enclosed in square brackets. ARPES measurements on TI nanoplates can directly detect the presence of the topological surface state. By performing electron transport experiments on nanodevices made of both topological insulators (TI) and topological crystalline insulators (TCI), one can see the surface states' two-dimensional and helical properties. Moreover, the characteristics of the superconductivity generated in TI nanostructures can be understood by performing experiments using Josephson junction devices [64]. Quantum interference experiments were done on nanostructures constructed of Cd3As2, 1T-MoTe2, and 1T-WTe2 to assess the surface transport parameters of Dirac and Weyl semimetals [[65], [66], [67], [68], [69]]. Fig. 2(a) shows a scanning electron microscope (SEM) image of the as-produced nanowires, which are 10 μm long and 50 nm–150 nm wide; Fig. 2(b) identifies the growth direction and single-crystal rhombohedral phase of the Bi₂Se₃ nanowires; Fig. 3(c) presents a schematic of the Bi₂Se₃ nanowire field-effect transistor (FET); and Fig. 4(d) displays a transmission electron microscope (TEM) image of the nanowire cross-section [70].

### 2.4 Spin qubits in nanowire

A quantum computer can be built by utilizing the spin of an electron or hole that is confined within a semiconductor quantum dot (QD) [71]. Methods for manipulating the spin qubit using magnetic and electric fields are suggested [[72], [73], [74], [75]]. The utilization of electric-dipole spin resonance (EDSR) for the electrical control of the spin qubit is favorable in physical implementations, despite the fact that managing the spin qubit is easier using magnetic fields [[76], [77], [78]].

## 3 Quantum gate operations

Prime factorization and the simulation of complex quantum systems are two significant computational challenges that beyond the capability of traditional computers. Quantum computers offer the potential to efficiently carry out these operations [79]. A digital quantum computer possesses a native gate set, which comprises the operations that may be physically executed in the hardware. Remarkably, finite collections of native gates have the capability to combine any operation with any required level of accuracy, thus establishing these gate sets as universal [80]. Maintaining coherence in quantum computing is a significant obstacle due to the inherent susceptibility to errors in all components of a quantum computer, such as physical qubits, gate operations, and measurements. The methods of quantum fault tolerance can remove this obstacle to large-scale quantum computation [81]. Some groups of fundamental operations are functionally complete in the older computers. It is possible to carry out each operation by combining and recombining such a set of procedures. For instance, the NAND gate is entirely functional on its own! The space of potential quantum operations is continuous, in contrast to the discrete set of operations used to express it in classical computers, hence the cardinals are not entirely equivalent. Nevertheless, a variety of gates can be used to approximate random quantum actions. In essence, quantum operators are what the quantum gates are [82]. Fig. 3 displays the Quantum gates and their associated operations.

### 3.1 Pauli gate

Nanoscale refers to a scale of measurement that is extremely small, typically on the order of nanometers. All-optical logic gate technologies are the preferable option for applications that involve all-optical computing and signal processing. All-optical quantum computing provides unparalleled processing power and the capacity to execute operations without any loss. The Pauli X, Y, and Z gates are essential quantum logic gates in the field of all-optical systems [83]. The limited bandwidth of the electronics systems is regarded as a barrier in the information processing process [84]. All-optical signal processing is a distinctive platform with diverse applications in logic units, telecommunications, and quantum computing. The reason for this is that it has inherent characteristics such as high velocity, low photon coupling, and an advantage in long-range transmission [85]. All-optical systems contain numerous passive components that can be employed to manipulate light, minimize heat generation, and improve fan-in and fan-out capabilities [86]. Historically, there have been multiple suggestions for optical logic gates, memory units, and other devices that utilize semiconductor optical amplifiers, electro-optic modulators, and non-linear materials. These sizable devices were restricted to functioning at speeds ranging from 93 to 94 gigabits per second (Gbps). Previously, numerous suggestions have been made for optical logic gates, memory units, and other devices that utilize semiconductor optical amplifiers, electro-optic modulators, and non-linear materials. These sizable devices were restricted to functioning at rates within the gigabits per second (Gbps) range [87].

### 3.2 Phase gate

Since the 1940s, a phased method has been employed in investment decisions for development, especially in large-scale projects related to mechanical and chemical engineering. According to a single account, there were a total of eight phases. In the last ten years, there has been significant advancement in circuit quantum electrodynamics (QED), a scientific discipline that employs microwave resonators or cavities and superconducting (SC) qubits. References [[88], [89], [90]] demonstrate that the circuit is very suitable for quantum information processing. Superconducting qubits are crucial in quantum information processing (QIP) because they can modify the gap between energy levels, have the potential for circuit scalability, and improve coherence times. Experimental data has shown a strong and consistent association between a superconducting qubit and a microwave resonator [[91], [92], [93]]. Furthermore, researchers have achieved the creation of a microwave resonator that exists in three dimensions, exhibiting a loaded quality factor of 3.5 x 10^7. Additionally, a coplanar waveguide microwave resonator has been developed, demonstrating a burdened quality factor of 10^6 [10^2]. Microwave photons in a high-quality factor microwave resonator or cavity can serve as a quantum data bus and a quantum memory due to their much longer lifespans compared to SC qubits [[94], [95], [96]].

### 3.3 Rotation gate

The Quantum-inspired Evolutionary Algorithm (QEA) created by Han and Kim has been widely and successfully employed in several industries. QEA encodes a solution individual using a quantum bit, which is represented by a pair of normalized probability amplitudes. Quantum bit coding offers a wide array of possibilities and represents the linear superposition of 0 and 1. QEA possesses the potential to accurately identify the global optimum with a high probability, even when employing a small population size [97,]. The quantum rotation gate (QRG) is the predominant quantum gate utilized in quantum error correction algorithms (QEA). There is a genetic quantum approach available for addressing knapsack issues. The QRG encompasses the definition and operational methodology, wherein the sole operational parameters are the directions and magnitudes of the rotation angles. The parameters are obtained using a lookup table [98]. QRG algorithms are frequently used in literature to implement dynamic rotation angles. Bin et al. introduced a quantum-inspired binary gravitational search method to tackle the challenge of incorporating wind power into thermal unit commitment. The QRG system is characterized by rotation angles that fluctuate based on the generation [99].

### 3.4 SWAP gate

It is important to consider the limited connectivity of many near-term quantum computers. One way to overcome limited connection is by incorporating swaps into the circuit, which allows for logical operations to be performed on qubits that are physically adjacent to each other. The problem of "routing via matchings" is commonly referred to as solving [100]. Experimental quantum computing is currently progressing towards achieving quantum supremacy, which refers to the stage where quantum computers can perform certain specialized tasks that are beyond the capabilities of even the most powerful classical supercomputers. Nevertheless, there is an additional technological milestone to be achieved: practical quantum supremacy. This would enable quantum computers to solve problems of significant value, irrespective of the method used to arrive at the solutions. Long-term progress in this area is probable, unless there are significant obstacles, due to the combination of efficient quantum algorithms with error correction that can be scaled up [101,102]. Presently, we possess Noisy Intermediate-Scale Quantum (NISQ) devices, which have minimal resources but have the potential to surpass classical devices in some scenarios. The interconnection of various technologies, including superconducting quantum processors, will be limited [103]. The majority of current quantum algorithms assume the existence of an abstract device that has unlimited connectivity, meaning it can perform a two-qubit gate operation between any pair of qubits. Considering that circuits can be assembled to any specific group of devices with a polynomial increase in the number of qubits and gates, this should be theoretically adequate. The presence of polynomial overheads is of practical importance and can determine whether a solution is possible or not on NISQ devices [104].

## 4 Entanglement engineering

Entanglement-based quantum science leverages the subtleties of quantum mechanics for applications such as metrology, sensing, and quantum computing [105]. In two well-known quantum theory paradoxes, the concept of quantum entanglement is present. For a long while, experiments meant to demonstrate the validity of quantum mechanics were particularly interested in entanglement [106]. Numerous nanowires are applicable in this field, such as free-standing silver nanowires that are even used to transmit plasmon quantum entanglement in the quantum region [107]. Several techniques for quantum entanglement engineering involve the use of nanowires Fig. 4.

### 4.1 Quantum dots

Semiconductor quantum dots that are incorporated into nanowires show great potential as suitable options for fulfilling the strict criteria of a "ideal" entangled photon source, which is necessary for the successful implementation of ambitious schemes in quantum information processing [108]. GaAs quantum dots in nanowires are a highly promising choice for achieving scalable quantum photonics. These devices can be adjusted to match the frequency of atomic transitions and possess outstanding optical characteristics [109]. An advantage of quantum dots (QDs) compared to other single quantum emitters is their compatibility with existing semiconductor technology. These variables have resulted in the current contention that they could evolve into the "optimal" origins of entangled photons [110]. Despite the interdot distance being smaller than the operating wavelength, it has been proposed that two quantum dots (QDs) can become entangled by interacting within the same cavity [111]. Entanglement between the quantum dots (QDs) arises spontaneously due to their shared interaction with the plasmonic nanostructures. There is no need for selected observations or deliberate manipulation of the dissipative environment to achieve this entanglement. Quantum information and computing rely on entanglement, a distinct quantum characteristic, which theoretically suggests the potential for actual "quantum plasmonics” [112]. Quantum dots have the ability to generate polarization-entangled photons through a process called the biexciton-exciton cascade [113].

### 4.2 Superconducting nanowires

Superconducting nanowire single-photon detectors, also known as SNSPDs, are extensively employed for the detection of photons throughout the visible and near-infrared spectrums. SNSPDs have demonstrated exceptional performance in the second scenario. Their system has achieved system detection efficiencies (SDE) above 90 % [114,115], dark count rates (DCR) below 1 count per second (cps) [116], count rates (CR) exceeding 1.5 billion counts per second (Gcps) [117], and precise timing with a temporal precision of less than 15 ps (ps) [118,119]. Detectors [120] have been effectively utilized in diverse applications, such as high-speed optical communication [121], time-of-flight ranging systems [122,123], and quantum information processing [124]. Superconducting nanowire single-photon detectors are becoming more popular in the disciplines of quantum optics and quantum communication due to their minimal temporal variation and excellent quantum efficiency in detecting low-energy photons [125].

### 4.3 Topological nanowire

Entangled multiphoton states are the essential building blocks for quantum computers, communications, and information. While researchers have successfully confirmed the topological protection of correlated and single photons through experiments, the observation of topologically protected entangled states has remained difficult [126]. The experimental achievement of the Majorana fermion (MF), a fermion that is self-conjugate and exhibits non-Abelian exchange statistics, has greatly increased the interest in topological quantum computation [127]. Although initially conceptualized in two dimensions, topological superconducting phases can also exist in one-dimensional systems, such as wire networks and nanowires, where braiding operations can be carried out. Compelling empirical data indicates that semiconductor nanowires have been employed to achieve a topological superconductor [128]. Presently, spin-orbit linked superconducting nanowires containing Majorana fermion zero modes (MZMs) are considered to be highly promising for the development of a topological qubit [129]. Precise management of electrostatics at wire junctions is essential for the manipulation and intertwining of non-Abelian Majorana zero modes, which are concentrated in semiconductor nanowires that have been proximitized. This is crucial for achieving topological quantum computation. This enables the implementation of anyonic fault-tolerant gate operations [130].

### 4.4 Photonic nanowire

Integrated photonics is a highly promising technology for generating entangled quantum states. This is because it requires little pump power, offers great stability, scalability [131,132], and has the advantage of being portable for distributed quantum networks [[133], [134], [135], [136]]. In order to conduct multiphoton studies using spontaneous parametric down-conversion (SPDC), it is important to maintain a low probability (p) of creating a single photon pair per pump pulse, often less than 0.1 [137]. This will effectively eliminate the undesired noise generated by the double-pair emission rate (∼p2). Optimizing the spatial and spectral characteristics of the pulsed SPDC is crucial in order to improve the efficiency of collecting photons into a single spatial mode. This will result in a higher count rate of entangled photons, while still maintaining the purity and indistinguishability of single photons. Nevertheless, the ability to gather all the necessary particles in previous studies involving multiphoton entanglement [138] was not enough to demonstrate entanglement with 10 photons [139].

### 4.5 Quantum sensing

Nanowires have been investigated for their potential use in quantum plasmonic sensing, among other quantum applications [[140], [141], [142], [143], [144], [145], [146]]. Utilizing plasmonic entanglement for sensing is particularly advantageous for sensitive systems such as photosensitive biological samples. In these cases, simply increasing the optical power to enhance sensitivity might cause optical damage to the specimens under study [147]. Quantum sensing is a rapidly advancing field with a broad spectrum of potential applications. This statement encompasses all quantum protocols that are capable of surpassing any classical method in terms of discrimination and estimation [148]. Table 1 provides a general overview of the characteristics, benefits, and difficulties of the many kinds of quantum sensors [149].

**Table 1:** Different types of quantum sensors [149].

Technology	Quantum features	Experimental conditions	Advantages vs classical systems	Challenges	Refs.
Nonphotonic quantum sensors	Spin qubits, neutral atoms, trapped ions	Multiple parameter measurements	High sensitivity, low noise	Decoherence, quantum projection noise	164
Remote target detection	Quantum illumination, quantum entanglement	Quantum interferometry	Enhanced signal-to-noise rat	Very fragile concerning optical loss	165
Quantum radar	Microwave quantum illumination	Quantum interferometry	Expose stealth targets	Lack of photon-microwave converters	166
Quantum spectroscopy	Quantum entanglement, single photons	Intensity correlation measurements	Beyond the shot-noise limit, approaching the ultimate quantum limit	Quantum decoherence	167
Quantum reading of optical classical memory	Quantum channel discrimination	Interferometer and single-photon source	Error-free, faster optical readers and denser memories	Using photon sources and detectors with very high efficiency	168

## 5 Quantum error correction and scalability

**Fig. 5:**
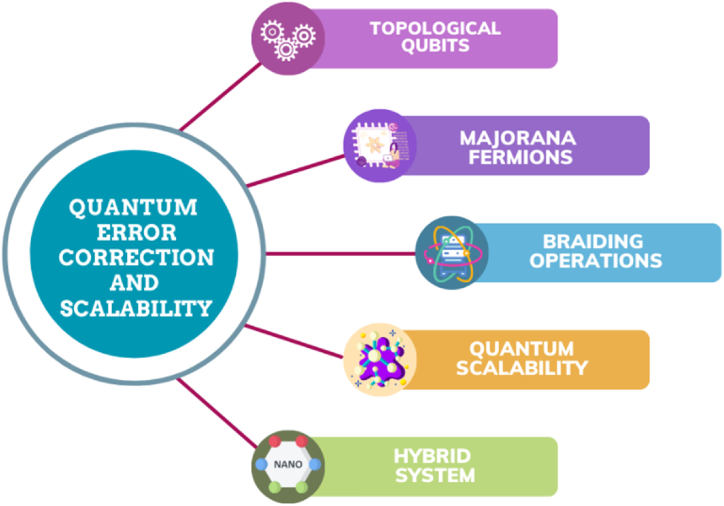
Quantum error correction and scalability methods.

Because of their special qualities, ultra-thin wires with widths on the order of nanometers are intriguing for applications in quantum computing. Nanowire can be used in quantum error correction and scalability. Quantum error correction and scalability can be divided into separate types. Fig. 5 shows some important methods of using nanowires in quantum error correction and scalability.

### 5.1 Topological qubits

A type of stabilizer error-correcting code with practical features is called a topological code. Their current status as the top contenders in the hunt for quantum error-correcting codes that can be included in a plan to create an experimentally implantable quantum computer is a direct consequence [150]. The primary obstacle to accomplishing quantum computation is handling quantum error. Because of the spatial separations between the boundary modes and anyons, topological qubits have inherent fault tolerance, which makes them valuable in this context. One of the most promising approaches in topological quantum computing is the study of Majorana zero modes, which arise as end modes of p-wave superconducting nanowires [151]. Topological qubits may become incoherent as a result of quasiparticle poisoning (QP), a solely fermionic mistake that modifies the fermion parity connected to Majorana degrees of freedom [152]. Superconducting qubits are known as "noisy intermediate-scale quantum computers" because of their sensitivity to noise, which increases inaccuracy with an increase in the number of qubits. As a result, one of their primary issues is scalability. This problem might be resolved by topological qubits because they are fault-tolerant topological quantum computers [153].

### 5.2 Majorana fermions

The possibility of utilizing Majorana fermions in fault-tolerant quantum computation is extremely fascinating [154]. A one-dimensional nanowire was subjected to a novel technique that involves the combination of spin-orbit interaction and Zeeman field to induce s-wave pairing and ultimately produce Majorana fermions. A system of this nature can be developed using the existing experimental techniques [155]. Qubits based on Majorana particles provide longer coherence lengths and improved fidelity for single-qubit Clifford gates through the process of braiding. In addition, they enable ancilla-free stabilizer measurements for quantum error correction [156], which is not possible with ordinary qubits. The Majorana fermion surface code is derived by employing the commuting Hamiltonians of two-dimensional interacting Majorana fermions with Z2 topological order [157]. Majorana zero modes, known for their nonlocal quantum information storage, are very resilient to noise and have been suggested as a key element in quantum computers [158].

### 5.3 Braiding operations

Decoherence poses a substantial impediment to the realization of large-scale quantum computing. Utilizing topologically protected non-Abelian anyons to implement fault-tolerant quantum computation is an appealing choice due to the inherent fault tolerance provided by topological protection. Zero-energy quasiparticles known as non-Abelian anyons can be created at the boundaries of a topological superconductor system. These quasiparticles are now referred to as Majorana zero modes (MZMs). By combining two Majorana zero modes (MZMs), it is feasible to attain quantum gates and a qubit that are protected by topology [159]. The use of two-dimensional quantum systems with anyonic excitations in the field of quantum information science is particularly intriguing because of its topological properties. The anyons offer inherent safeguards for maintaining quantum coherence. These systems that are arranged in a topological manner have the ability to achieve quantum gates by manipulating anyons through braiding. Anyons, being intrinsically topological, are resistant to errors, making them highly reliable. Additionally, there is a degeneracy in the ground-space that is contingent upon the topology of the system. Ultimately, these systems are not affected by small changes in their immediate surroundings [160].

### 5.4 Quantum scalability

**Fig. 6:**
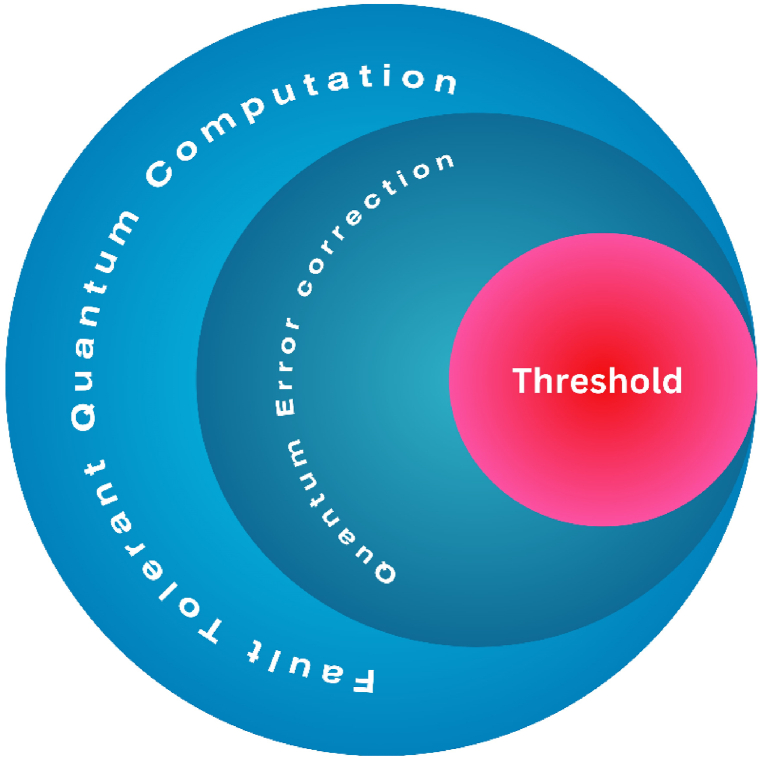
The scalability hierarchy. The foundation of effective quantum error correction (QEC) is the threshold. One essential and essential element in the field of QEC is the threshold. It serves as a crucial reference point for assessing how well QEC methods rectify faults that arise in quantum systems. QEC is not feasible, if not impossible, to deploy in practice without reaching a high enough threshold. Crossing the threshold, however, is the moment at which QEC starts to effectively repair more mistakes than we cause. Shir's algorithm and quantum simulation are two examples of actual quantum applications that need great precision and reliability. The implementation of QEC may allow Fault-Tolerant Quantum Computation (FTQC). Thus, achieving a high threshold not only guarantees QEC's viability but also opens the door to fault-tolerant quantum computation (FTQC).

Scalability is an essential element that must be present in any forthcoming computing system. The Riel group has recently demonstrated the ability to create patternable ballistic InAs NW crosses on silicon using template-assisted growth [161]. An important challenge that needs to be addressed is the capacity of solid-state spin qubits to couple and entangle across long distances. This feature is essential for implementing fault-tolerant quantum error correction techniques and for building scalable quantum computer architectures [162]. The Majorana qubit is expected to surpass other potential platforms for quantum computing in terms of scalability due to its great fidelity [163]. Quantum computers are essential for achieving qualitative relevance in scalable quantum algorithms [164]. The HHL (Harrow, Hassidim, Lloyd) algorithm [165], Shor's factorization algorithm, quantum simulation [166], and other quantum algorithms that have demonstrated increased speed are instances of algorithms for solving linear systems of equations. However, these algorithms can only be implemented when utilizing thousands of qubits that are nearly perfect. Given the unlikelihood of physical error rates becoming extremely low in the near future, it is necessary to have fault tolerance and quantum error correction (QEC). The threshold value functions as the essential metric for practical QEC implementations. Logical qubit errors will be suppressed to zero and the error rate will stay below a predetermined threshold when the error rate of physical qubits falls below this number. At this point, QEC kicks in and corrects more errors than we do. Conversely, if the error rate of physical qubits exceeds this threshold, the logical qubit errors will grow until they reach infinity. Therefore, the maximum permissible amount of physical error per gate for each qubit must be as large as possible at the threshold value to ensure the efficacy of QEC [167] (Fig. 6).

### 5.5 Hybrid system

For a while now, there has been a search for a clear explanation of how quantum-classical hybrid systems behave. This search aims to achieve several objectives, such as understanding how mesoscopic systems work, finding a way to combine quantum theory and gravity, and explaining how quantum measurements occur [[168], [169], [170], [171], [172], [173]]. The utilization of atomic resonance has been employed to precisely control and adjust the emission frequency of quantum dots, resulting in the creation of a highly stable source of single photons. Hybrid systems enable the operation of several emitters at the exact same wavelength, which helps overcome a major challenge in quantum information processing and communication [174]. Nanowires have the potential to form hybrid quantum systems by integrating various qubits or quantum technologies. The hybrid nanowire-based technique is advantageous as it eliminates the need for exact positioning of individual nanowires by utilizing Josephson junctions that are integrated inside a two-dimensional structure as the nonlinear component. Superconducting qubits [175] are a branch of research that focuses on scalability as one of its key objectives.

## 6 Applications and algorithms

Processing massive amounts of data is now possible because of advancements in computing technology. The ability of quantum computing (QC) to do difficult jobs significantly more quickly than traditional computers has been demonstrated [176]. Now that a large number of people have started to investigate and engage with quantum computers, research towards useable quantum algorithms has been stimulated, attracting interest from the academic, governmental, and corporate worlds [177]. The quantum applications cover fields like data encoding, compilers, or the detection of entanglement, identification of defective gates, and quantum machine learning that are connected to quantum circuits [178].

### 6.1 Applications

**Fig. 7:**
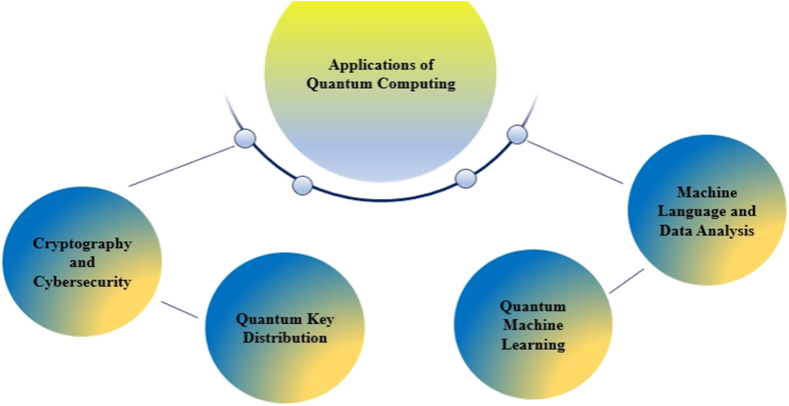
Applications of quantum computing.

Quantum computing revolutionizes cryptography, optimizes complex systems, and accelerates material and drug discovery. It enhances AI by improving machine learning, driving advancements across scientific and industrial fields. Fig. 7 shows the applications of quantum computing.

#### 6.1.1 Cryptography and cybersecurity

##### 6.1.1.1 Quantum key distribution

Quantum key distribution (QKD) allows for the secure distribution of keys that are guaranteed to be safe from an information-theoretical standpoint [179,180]. The BB84 protocol, first introduced by Bennett and Brassard in 1979 and then published in a computer conference proceeding in 1984, is widely acknowledged as the groundbreaking Quantum Key Distribution (QKD) technology [181]. The B92 protocol is a cryptographic modification of the BB84 protocol, despite their distinct physical characteristics [182]. Quantum Key Distribution (QKD) is a cutting-edge technique that employs the laws of quantum physics to securely transmit random secret keys between two users, even in the presence of an unauthorized listener. The creation of Quantum Key Distribution (QKD) relies primarily on the no-cloning theorem and Heisenberg's uncertainty principle [183].

**Fig. 8:**
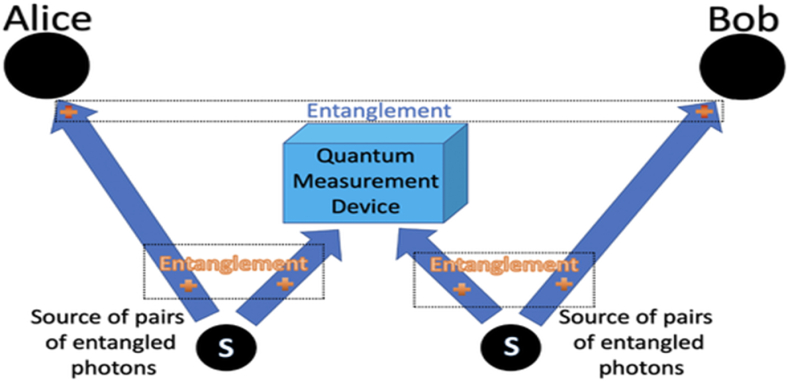
Quantum Key Distribution [185].

Even in the scenario of Fig-8, a possible eavesdropper with limitless computer capacity, quantum key distribution (QKD) enables Alice and Bob to successfully extract a string of symmetric keys and finish the perfectly secure communication of one secret at a time [184].

#### 6.1.2 Machine language and data analysis

##### 6.1.2.1 Quantum machine learning

Quantum machine learning (QML) is a novel paradigm of parallel computation that combines quantum or conventional computational systems and algorithms with network theory. Its purpose is to enhance computation speed and existing capabilities of NISQ-era quantum technology. Quantum computers have the ability to run at far faster speeds than conventional computers, according to the phenomenon of quantum parallelism. However, as traditional algorithms may not be sufficient, it is necessary to develop novel quantum algorithms. Shor's approach is capable of efficiently factoring large integers by utilizing quantum parallelism [186]. QML finds applications in diverse domains such as image processing, computational biology, bioinformatics, particle physics, communication networks, and privacy protection. QML methods, such as amplitude amplification, provide a fair balance between temporal complexity and traditional approaches for problems related to pattern classification and identification [187]. Table 2 presents a comprehensive overview of the several uses of quantum computing.

**Table 2:** Applications of quantum computing.

Applications of Quantum Machine Learning	Description
Quantum Data Analysis and Classification	faster than conventional techniques in searching an unsorted database.
Quantum Data Regression	quicker than standard methods for searching an unsorted database.
Quantum Support Vector Machines Enhancing	discovering the best decision boundaries and translating data to quantum states to improve SVMs.
Quantum Generative Models	encoding and producing complicated quantum states for the simulation and discovery of materials.
Quantum Neural Networks	neural networks with quantum circuit integration for faster inference and training.
Quantum Boltzmann Machines	using quantum versions for unsupervised learning, data production, and optimization.
Quantum Data Clustering	Quantum parallelism speeds up k-means clustering for data processing.
Quantum Dimensionality Reduction	reducing the number of dimensions in quantum data for analysis and display.
Quantum Anomaly Detection	finding anomalies or outliers in large datasets using quantum machine learning.
Quantum Optimization	optimizing activities, including portfolio management, using quantum algorithms.
Quantum Chemistry and Drug Discovery	speeding up molecular behavior simulations for drug development and materials research.
Quantum-enhanced Reinforcement Learning	improving decision-making through the use of quantum algorithms in reinforcement learning.
Quantum Data Compression	investigating practical techniques for quantum data storage and compression.

### 6.2 Algorithms

**Fig-9:**
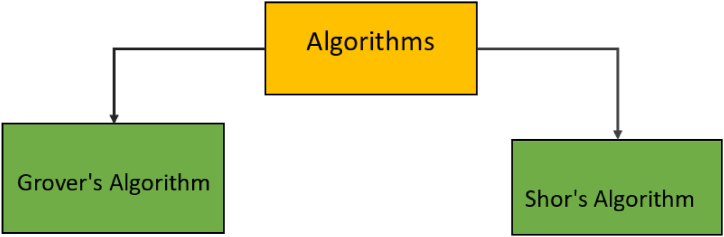
Algorithms of quantum computing.

Quantum computing algorithms exploit quantum phenomena like superposition and entanglement to perform calculations exponentially faster than classical algorithms, revolutionizing cryptography, optimization, and simulation tasks across industries. Fig. 9 illustrates the algorithms of quantum computing.

#### 6.2.1 Shor's algorithm

Shor's algorithm is the most illustrative and engaging quantum algorithm [188]. In 1994, Shor developed Shor's method, proving that quantum computers are capable of polynomial fast prime factorization. The fastest traditional method's time complexity is exponentially increased by the first quantum algorithm, which significantly raises interest in and funding for quantum computing in the academic world [189]. This computation can be done significantly more quickly by quantum computers thanks to Shor's algorithm, a quantum algorithm [190].

##### 6.2.1.1 Shor's algorithm in discrete logarithm problems

Quantum computers have demonstrated that solving the discrete logarithm problem is not challenging, according to Shor's Algorithm [191]. Shor's method successfully solves the discrete logarithm problem (DLP) by using the inherent commutativity structure of the group [192]. The discrete logarithm (DL) problem and its related challenges have been fundamental cryptographic primitives for a significant period of time in the era before quantum computing [DH76, Gam 85]. The emergence of quantum computing has had a profound effect on the field of cryptography in the post-quantum era. Due to Shor's approach [Sho94], the problem of DL (and integer factoring) may now be efficiently solved in quantum polynomial time. As a result, numerous cryptographic schemes that depend on the DL problem are no longer secure against fully operational quantum computers [193].

#### 6.2.2 Grover's algorithm

Grover's algorithm is a quantum method that may be used to search an unsorted database or find a specific item in a list of unstructured data. It was created by Lov Grover in 1996, and today is recognized as one of the most important quantum algorithms [194]. Well-known quantum algorithm Grover's search can quicken the thorough key search against symmetric key cryptography [195]. The likelihood of receiving accurate results when using Grover's technique to search an unordered database typically declines as the number of marked items rises [196]. Grover's Search was a ground-breaking algorithm when it was first developed, and its underlying technique of amplitude amplification has served as a foundation for several other algorithms and patterns for decoding information contained in quantum states [197].

## 7 Implications and potential impact

Quantum technologies have experienced tremendous progress, with important ramifications [198]. Although its effects are still being studied, quantum computing has the potential to transform a wide range of fields in science and industry.

### 7.1 Cryptography in quantum computing

There is no denying that modern society is based on technical breakthroughs, notably in the area of electronic communications. The science of cryptography is one of the most crucial areas of study in information technology since data transmission and storage require secrecy, integrity, authenticity, and non-repudiation. The act of protecting data from third-party adversaries while it is in transit or being stored, known as cryptography, is etymologically derived from the Greek terms for concealed and writing [199]. The field of quantum information science has perhaps seen the quickest growth in quantum cryptography. Regularly new theoretical protocols are developed, security proofs are improved, and trials gradually go from proof-of-concept lab tests to real-world deployments and technology prototypes [200]. The security of internet communication, automobiles, and implanted medical devices depends on cryptography [201]. Traditional cryptography either assumes that no one can answer a particular challenging mathematical issue in a reasonable period or uses information theory justifications. Instead, quantum cryptography is based on the basic principles of quantum physics [202].

**Fig. 10:**
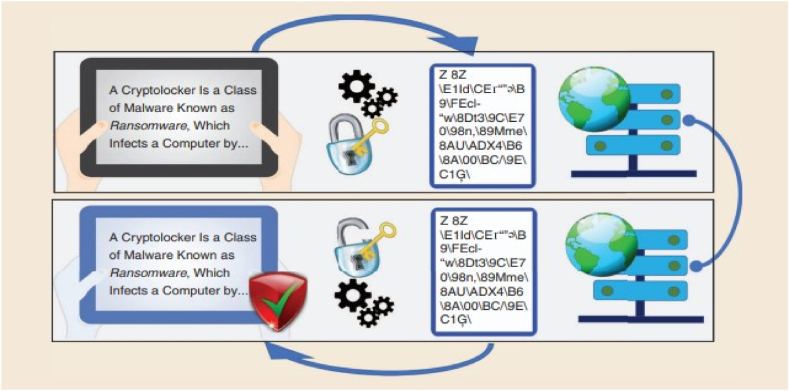
Encryption decryption process in cryptography [204].

Cryptography, often known as cryptology, refers to the practice of using hidden or secret writing to enable secure communication. In order to establish secure communication, both parties involved must reach a consensus on a specific encryption mechanism for encoding and decoding the data. After selecting a method for encrypting and decrypting Fig. 10 data, the communicators exchange a secret key, which is also referred to as the encryption key [203].

### 7.2 Climate change

To determine how or where emerging quantum technologies may be applied to slow down the pace and impact of climate change, the Climate Initiative brings together the research communities at the intersection of quantum and climate science, involving academia, government, and industry [205].

#### 7.2.1 Impact of quantum computing on climate change

##### 7.2.1.1 Renewable energy

**Fig. 11:**
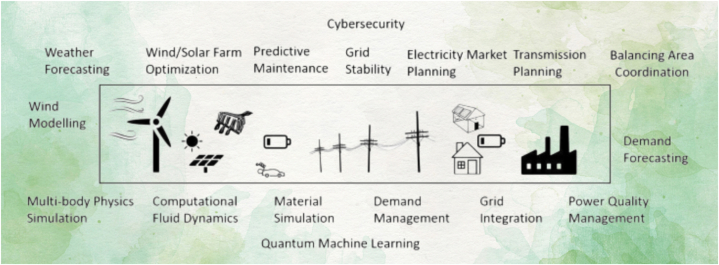
Quantum computing in renewable energy [209].

Quantum computers has an extraordinary computational capacity that could potentially lead to a disruptive revolution in the field of renewable energy. The combination of quantum computing's computational power and optimization capabilities, along with efficient algorithms that can greatly speed up renewable energy process modeling, is expected to greatly change the way we explore renewable energy sources [206]. As renewable resources are accepted and ramped up, the electrical system will encounter significant challenges during the coming decades. These issues include the requirement to coordinate more dispersed resources and take weather-dependent power patterns into account. The present issues with renewable energy might be made worse by quantum computing. The challenges that present technology may face in the future may be solved by quantum computing [207]. Quantum computers are highly successful in handling the intricate problems associated with optimizing renewable energy sources. Due to its superior performance in terms of objective function value and processing time, the quantum method provides a viable approach to developing cost-effective and durable renewable energy solutions. The scalability of quantum computing and its dependence on quantum phenomena provide more evidence of its revolutionary impact on strategies for optimizing renewable energy. This opens up possibilities for a safer and more ecologically friendly future of energy [208]. Fig. 11 illustrates the utilization of quantum computing in the renewable energy sector.

## 8 Challenges and future directions

### 8.1 Challenges

Finding a means to use gadgets without having accurate knowledge of their physical properties is the difficulty of quantum computing [210]. If a large-scale implementation of quantum computing can be created, it has the potential to provide exponentially more powerful processing. The construction, verification, and architectural hurdles of creating a large-scale quantum computer are numerous [211].

The First Working Seminar on Quantum Software Engineering (WSQSE 2022) was held in Innsbruck, Austria, on December 15 and 16, 2022. 33 researchers and practitioners in software engineering and quantum computing attended the two-day conference [212]. The software business has faced unforeseen hurdles as a result of the present technological revolution. Software engineering is about to undergo a revolution thanks to the development of quantum computing (QC) technologies during the past several years. The evaluation and prioritization of QC concerns in the software sector, however, are still understudied, poorly understood, and dispersed [213]. Affecting all facets of software engineering is quantum computing. In reality, updating SWEBOK (Software Engineering Body of Knowledge) to include quantum concerns should be done in the majority of the 14 categories. Since the early 1900s, quantum mechanics has been studied. Today, we have multiple quantum computers that incorporate various technological levels multiple algorithms have also been presented, and several programming languages are also accessible. Therefore, it is now time to suggest and validate software engineering methodologies to usher in a new era of peak achievement for quantum software engineering [214,215].

### **8.2** Future directions

A whole new and rich paradigm for investigation has been given to information theorists and computer scientists. In the broadest sense, any physical theory—not just quantum mechanics—can serve as the foundation for a theory of information processing and transmission. These research efforts might one day provide information processing devices that are significantly more advanced than current computer and communications systems, with both advantages and disadvantages for society as a whole. The obstacles that come with quantum processing and quantum information are many, but the long-term benefits that these concepts bring to physics are probably a bit more subtle [216]. Modern computers are built on the principles of classical physics, both in theory (Turing machines) and in practice (PCs, HPCs, laptops, tablets, cellphones, etc.). They are constrained by localization (operations only have local effects) and the conventional restriction that systems may only exist in a single state at a time. Modern quantum physics, on the other hand, teaches us that the universe operates rather differently. During its evolution, a quantum system may show interference effects and be in a superposition of several distinct states at once [217]. Finding effective solutions for a variety of problems that are not known to be N P-complete and do not have a known effective classical solution is one of the key topics of research in quantum algorithms. This is the Graph Isomorphism issue, which involves determining if two graphs are isomorphic. Finding algorithms that more accurately imitate quantum physical systems is a key area of research for quantum algorithms. Quantum complexity research is still in its infancy [218].

## 9 Conclusion

The review study highlights the difficulties and barriers that currently hinder the complete achievement of the potential of quantum computing. Major obstacles arise from the need for efficient quantum error correction, specifically in relation to topological qubits that depend on non-traditional particles such as Majorana fermions. To achieve quantum scalability, it is necessary to coordinate qubits in hybrid systems that combine different technologies, including topological qubits and superconducting circuits. Innovative methods such as braiding Majorana fermions are being employed to tackle the ongoing problem of decoherence, which is crucial for the advancement of error-protected logical qubits. To fully harness the revolutionary potential of quantum computing in scientific, technological, and computational fields, it is essential to overcome challenges related to decoding mitigation, error correction, and hardware scalability.

Quantum computing's arrival holds great potential and represents a fundamental change with significant implications for multiple industries. The assessment emphasizes the profound impact on cryptography, which requires major changes to mitigate the risks posed by quantum algorithms. Quantum simulations provide accurate modeling for addressing climate change, while expediting research on materials and optimization for renewable energy holds the potential for transformative advancements in the energy industry. The interdisciplinary character of quantum computing, which integrates principles from quantum physics and materials science, fosters innovative approaches in environmental research and the development of sustainable technology. The review highlights the undeniable potential of quantum computing to revolutionize industries and reshape technological landscapes. It emphasizes the need for further research into fault-tolerant systems, novel qubit technologies, and optimized quantum algorithms, despite the existing challenges.

## Date availability

The authors confirm that the data supporting the findings of this study are available within the article.

## CRediT authorship contribution statement

**Mariam Akter Mimona:** Writing – original draft, Visualization, Supervision, Resources, Formal analysis. **Md Hosne Mobarak:** Writing – review & editing, Supervision, Resources, Formal analysis, Data curation, Conceptualization. **Emtiuz Ahmed:** Writing – original draft, Visualization, Resources. **Farzana Kamal:** Writing – original draft, Visualization. **Mehedi Hasan:** Writing – review & editing, Data curation.

## Declaration of competing interest

The authors declare that they have no known competing financial interests or personal relationships that could have appeared to influence the work reported in this paper.
